# Structural equation models to estimate risk of infection and tolerance to bovine mastitis

**DOI:** 10.1186/1297-9686-45-6

**Published:** 2013-03-06

**Authors:** Johann Detilleux, Léonard Theron, Jean-Noël Duprez, Edouard Reding, Marie-France Humblet, Viviane Planchon, Camille Delfosse, Carlo Bertozzi, Jacques Mainil, Christian Hanzen

**Affiliations:** 1 Department of animal production, University of Liège, Liège, 4000, Belgium; 2 Large Animal Clinics, University of Liège, Liège, 4000, Belgium; 3 Department of parasitic and infectious diseases, Faculty of Veterinary Medicine, University of Liège, Liège, 4000, Belgium; 4 Association Wallone de l’Elevage asbl, 4 rue de Champs Elysées, Ciney, 5590, Belgium; 5 Agriculture and Natural Environment Department, Farming systems, territories and information technology unit, Rue de Liroux 9, Gembloux, 5030, Belgium; 6 Production and Sectors Department, Animal nutrition and sustainability unit, Rue de Liroux 8, Gembloux, 5030, Belgium

## Abstract

**Background:**

One method to improve durably animal welfare is to select, as reproducers, animals with the highest ability to resist or tolerate infection. To do so, it is necessary to distinguish direct and indirect mechanisms of resistance and tolerance because selection on these traits is believed to have different epidemiological and evolutionary consequences.

**Methods:**

We propose structural equation models with latent variables (1) to quantify the latent risk of infection and to identify, among the many potential mediators of infection, the few ones that influence it significantly and (2) to estimate direct and indirect levels of tolerance of animals infected naturally with pathogens. We applied the method to two surveys of bovine mastitis in the Walloon region of Belgium, in which we recorded herd management practices, mastitis frequency, and results of bacteriological analyses of milk samples.

**Results and discussion:**

Structural equation models suggested that, among more than 35 surveyed herd characteristics, only nine (age, addition of urea in the rations, treatment of subclinical mastitis, presence of dirty liner, cows with hyperkeratotic teats, machine stripping, pre- and post-milking teat disinfection, and housing of milking cows in cubicles) were directly and significantly related to a latent measure of bovine mastitis, and that treatment of subclinical mastitis was involved in the pathway between post-milking teat disinfection and latent mastitis. These models also allowed the separation of direct and indirect effects of bacterial infection on milk productivity. Results suggested that infected cows were tolerant but not resistant to mastitis pathogens.

**Conclusions:**

We revealed the advantages of structural equation models, compared to classical models, for dissecting measurements of resistance and tolerance to infectious diseases, here bovine mastitis. Using our method, we identified nine major risk factors that were directly associated with an increased risk of mastitis and suggested that cows were tolerant but not resistant to mastitis. Selection should aim at improved resistance to infection by mastitis pathogens, although further investigations are needed due to the limitations of the data used in this study.

## Background

Public concern about farm animal welfare has steadily grown during recent years and scientists have searched methods to improve the ability of animals to defend themselves against pathogens.

One method is to select as reproducers, animals with the highest ability to fight infection. Indeed, it is well established that this ability varies substantially among and within breeds and is at least partly under genetic control [1]. The ability to fight infection may be characterized by two mechanisms: resistance, i.e., the ability to limit the parasite burden, and tolerance, i.e., the ability to limit the damage caused by a given parasite burden [2]. Operationally, resistance is typically measured by the number of parasites per host or per unit of host tissue [2,3] while tolerance is defined as the slope of a regression of host performance against infection intensity [2,3].

Mechanisms of resistance and tolerance can be further differentiated into direct and indirect mechanisms. Resistance traits are ‘direct’ when they reduce pathogen transmission by contact (resistance to infection) and ‘indirect’ when they reduce pathogen growth rate once infection has occurred, through the establishment of an immune response (resistance to disease). Tolerance traits are direct when they aim at reducing damage inflicted by the pathogen and indirect when the damage is caused by the immune response [4]. The distinction between these traits is important when determining selection objectives because they are predicted to have different evolutionary effects on pathogens and hosts [3,4] and they have been found to be negatively genetically correlated in plants and mice [2]. One way to test whether mechanisms are direct or indirect is to use structural equation models (SEM). These are multiple-equation regression models in which the response variable in one regression equation can appear as an explanatory variable in another equation. Variables can influence one-another reciprocally, either directly, or indirectly. A direct effect occurs if an explanatory variable influences the response variable directly, i.e., with no variables in the pathway between explanatory and response variables, and an indirect effect occurs when the influence of the explanatory variable on the response variable is mediated by one or more intervening variables. The sum of direct and indirect effects is the total effect [5].

The SEM can also be used to estimate the risk of infection, which is necessary to compare levels of direct resistance of animals placed in natural conditions. Indeed, the number of parasites in resistant animals living in an infected environment may be identical (or even higher) to the number detected in susceptible animals located in a clean environment. Therefore, for a fair evaluation, it is necessary to compare animals with the same opportunity or risk of encountering the pathogen. Unfortunately, estimating this risk of infection is not possible in field studies, since detailed and expensive epidemiological and laboratory data are needed, such as structures of contact between hosts and time data on when infection enters the population. An alternative is to characterize the risk of infection in each herd based on management practices known to influence it significantly and to classify herds into categories, from ‘high-risk’ to ‘low-risk’, based on these management practices. Herd management practices known to influence the risk of infection are numerous. For example, in bovine mastitis, Dufour et al. [6] identified more than one hundred such management practices. Besides being too numerous to be all surveyed on a routine basis, these practices are interrelated. One way to reduce the complexity of such situations is to use SEM with latent variables. A latent variable is a variable that cannot be measured but is inferred from one or more observed variables [5]. Management practices related to the risk of infection can then be aggregated in a few numbers of latent variables and alternative hypotheses about the relationships between latent variables and risk of infection can be tested.

The objective of this paper was to apply SEM to bovine mastitis. Specifically, we used SEM to (1) identify management practices that are directly related to the risk of infection in dairy herds of the Walloon region of Belgium and (2) estimate direct and indirect levels of tolerance of cows located in these herds.

## Methods

### Risk of infection

Data were from a random stratified sample of 345 dairy farms surveyed between January 2006 and October 2007 in the Walloon region of Belgium (project OSaM ‘Observatoire de la Santé Mammaire’). Farm characteristics and measures of herd prevalence were recorded for each farm. A complete description of animals and measurements can be found in Detilleux et al. [7]. Briefly, measures of mastitis prevalence (n = 6) included herd average of individual somatic cell count (SCC), herd somatic cell score (SCS), number and percentage of cows with SCC above 400 000 cells, and of cows milked aside because of high SCC. Farm characteristics (n = 35) consisted of herd demographics, productive and reproductive indicators, feeding procedures, types of housing, strategies of mastitis prevention and treatment, and milking methods.

We considered 28 latent variables in the first equation of the SEM, often called the ‘measurement’ part of the SEM. Each latent variable covered observed variables that were significantly associated with each other (one-to-one association; p ≤ 0.10). The first latent variable (η_1_, called ‘MAM’) included all six measures of mastitis frequency. The second latent variable (η_2_, called ‘AGE’) covered herd parity, age, and percentage of heifers in the herd. The third latent variable (η_3_, called ‘PROD’) included milk production, protein and fat percentages, and total milk quota. The fourth latent variable (η_4_, called ‘NUM’) covered the number of lactating and dry cows, and the fifth included time to prepare the udder, delay in installing the machine and the practice of hand washing after milking (η_5_ or ‘MLK’). The remaining 23 latent variables corresponded to the remaining 23 observed variables because no one-to-one associations were found among them. In the second equation of the SEM, the ‘structural’ part of the SEM, we modeled the links among the 28 latent variables (η). The premises for constructing this part of the SEM were that all latent variables could affect MAM and be themselves affected by latent variables other than MAM and by the variable itself.

In matrix notation, the SEM is:

y=Λη+υ,

η=Bη+ζ,

where **y** is the (41 X 1) vector of observed variables, **η** is the corresponding (28 X 1) vector for latent variables, and **υ** and **ζ** are the corresponding vectors of error terms. It is assumed E(**υ**) = **0**, var(**υ**) = **Θ**, E(**ζ**) = **0**, and var(**ζ**) = **Ψ**. Elements (λ) of **Λ** are partial regression coefficients relating latent variables to the observed variables, while elements (β) of **B** connected latent variables among them (direct and indirect effects). All computations were done with the LISREL^®^ program 2.8 [7], using the maximum likelihood–mean adjusted method [8]. Following [9], we considered as final, the model that had the superior fit. This model was the model for which the root mean square error of approximation (RMSEA) ≤ 0.05, the Goodness of Fit Index (GFI) ≥ 0.9, the Normed Fit Index (NFI) ≥ 0.9, and for which partial regression coefficients (β) were not different from null (p < 0.05).

### Direct and indirect tolerances

Data were from cows belonging to four farms surveyed by the project OSAM. During April 2012, two surveyors collected milk samples from 346 cows, immediately before evening milking. They cleaned teat ends with alcohol swabs and allowed them to dry. They discarded the first few streams and collected milk samples in sterile plastic tubes. Samples were immediately cooled, transported in cool bags to the Bacteriology laboratory of the Veterinary Faculty in Liège, and stored overnight at 4°C. In the absence of macroscopic alteration of the milk, one mL from each quarter of a cow were pooled and 100 μL were inoculated onto Columbia base agar (Merck-VWR, Belgium) plates supplemented with 5% bovine blood that were incubated overnight at 37°C.

Milk samples with less than 100 CFU/mL of one/two or of several different colony types were marked as ‘negative’ or ‘contaminated’, respectively. Samples with over 100 CFU/mL were marked as ‘positive’ if a maximum of two types of colonies were detected. Samples with over 100 CFU/mL and more than two colony types were also marked as ‘positive’ (but contaminated) if one colony type had counted for over 100 CFU/mL. Milk samples with macroscopic alterations and from each quarter of the ‘positive’ cows were individually inoculated onto the same blood agar plates and incubated overnight. Growth results were analyzed as described above. Counts from duplicate plates were averaged and CFU/mL were recorded as total bacterial infective dose for each quarter. Colonies from positive samples were identified to the following groups according to Gram staining, production of catalase and/or oxidase, production of haemolysis on blood agar plates and growth on selective agar plates (Table 1): Gassner (Merck-VWR, Belgium) for lactose fermentation by enterobacteria, Chapman (Merck-VWR, Belgium) for staphylococci growth and modified Edwards Medium (Oxoid, Belgium) with 5% bovine blood for esculin hydrolysis by streptococci/enterococci [10].

**Table 1:** Growth characteristics of the major and minor mammary gland pathogens^*^

Properties^**^	**1**	** 2**	**3**	**4**	**5**	** 6**	**7**	** 8**	**9**	**10**	**11**
Staphylococci	+	Cocci	+	**-**	+	β, **-**	**-**	NR^***^	+	**-**	NR
Streptococci	+	Cocci	**-**	**-**	+	α, β, **-**	**-**	NR	**-**	+	+, **-**
Enterococci	+	Cocci	**-**	**-**	+	α	**-**	NR	**-**	+	+
Enterobacteria	**-**	Short rods	+	**-**	+	α, β, **-**	+	+^****^, **-**	**-**	**-**	NR
Pseudomonads	**-**	Short rods	+	+	+	β^*****^, **-**	+	**-**	**-**	**-**	NR
Corynebacteria	+	Short rods	+, -	**-**	+	β, **-**	**-**	NR	**-**	**-**	NR
Bacilli	+	Long rods	+	**-**	+	β, **-**	**-**	NR	+, **-**	**-**	NR

^*^Presence of strict anaerobes and mycoplasmas was not assessed; ^**^properties are 1 = Gram staining, 2 = morphology, 3 = catalase production, 4 = peroxidase production, 5 = growth on Columbia blood agar, 6 = haemolysis on Columbia blood agar, 7 = growth on Gassner agar, 8 = lactose fermentation on Gassner agar, 9 = growth on Chapman agar, 10 = growth on Edwards medium, 11 = esculin hydrolysis on Edwards agar; ^***^NR = not relevant; ^****^also named coliforms: lactose-fermenting species of *Escherichia*, *Klebsiella*, *Enterobacter* genders; ^*****^due to the production of pigments the haemolysis zone can appear green to blue-green.

For statistical analyses, only the genus of major or minor mammary gland bacteria (Table 1) was taken into account. We computed udder-composite CFU as the sum of CFU of all bacterial species and all quarters, and restricted our study on direct and indirect tolerances to cows with udder-composite CFU > 0. We extracted data on udder-composite SCC (SCC = n cells/10^3^ per mL) and milk yield (= kg*10) from the regional milk-recording database from the date closest to the date when milk samples were collected. We computed bulk tank SCC by weighting individual SCC of all cows present in the herd by their milk production. We log-transformed SCC and CFU (base 2) so their distributions were closer to normality. Next, we constructed latent variables (denoted SCC*, CFU*, and milk*) for log(SCC), log(CFU) and milk yield to translate the fact that observed values were not error-free. We postulated four different SEM to analyze the possible interrelations between SCC*, CFU* and milk*, considering milk* as the end-point. Possible causal relations between SCC* and CFU* that were investigated were: (a) CFU* and SCC* are unrelated (null model), (b) CFU* influences SCC* (bacterial infection elicits an immune response), and (c) SCC* influences CFU* (immune response impacts bacterial infection). Each model included also the potential effects of bulk tank SCC, month in milk (fourth degree polynomial) and parity. In matrix notation, the SEM is:

y=Λη+υ,

η=Bη+Γξ+ζ,

where **y** is the vector of observed CFU, SCC and milk yield; **η** is the corresponding vector for latent variables (CFU*, SCC*, MILK*); **ξ** is the vector for the effects of bulk tank SCC, parity and month in milk; **υ** and **ζ** are the corresponding vectors of error terms, with E(**υ**) = **0**, var(**υ**) = **I**, E(**ζ**) = **0**, and var(**ζ**) = **Ψ**. Elements of **υ** and **ζ** were assumed independent. We performed statistical analyses with SAS9.1 using the CALIS procedure [11]. Parameters and effects (direct, indirect and total) were estimated by maximizing the likelihood of the data. Standard errors for effects were obtained by boostrapping the sample cows (1000 samples). Fit criteria included RMSEA, GFI and NFI [12].

## Results

### Risk of infection

**Figure 1:**
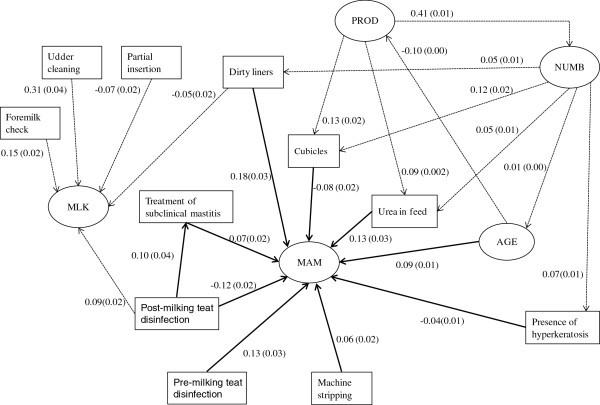
Final structural model describing the direct (straight line) and indirect (dashed line) links between the latent state of udder health (MAM) and risk factors.

Goodness-of-fit statistics (RMSEA < 0.01, NFI = 0.95, and GFI = 1.0) indicated that the final SEM closely fitted data. Standardized estimates of elements of **Λ** (measurement model) were close to or above 0.5 and significantly different from 0 at the 0.01 level, suggesting that the observed measurements were valid indicators of their corresponding latent variables [7]. Standardized estimates of elements of **B** (structural model) retained in the final SEM are given in Figure 1. Only nine herd characteristics (AGE, addition of urea in the rations, presence of dirty liners, cows with hyperkeratotic teats, machine stripping, pre- and post-milking teat disinfection, and housing of milking cows in cubicles) were directly and significantly related to MAM. The three most influential variables increasing MAM were the presence of dirty liners, followed by the addition of dietary urea, and the practice of pre-milking teat disinfection. In contrast, post-milking teat disinfection (the only variable with a coefficient below −0.10) was negatively associated with MAM, with a direct standardized link of −0.12 (SE = 0.02). Post-dipping was also indirectly and positively associated with MAM through its effect on the intervening variable ‘Treatment of subclinical cases of mastitis’. Latent variables PROD and NUMB were indirectly rather than directly associated with MAM, through their effects on some of the practices directly associated with MAM.

### Direct and indirect tolerances

Averages over all infected cows (28%) of CFU, SCC and milk yield were 1108 268 (SE = 98 940) CFU/mL, 631 674 (SE = 62 566) SCC/mL and 28.85 (SE = 0.55) kg/day. For the remaining uninfected cows (63%), averages were 310 045 (SE = 28 073) SCC/mL and 26.78 (SE = 0.31) kg milk. Samples from the remaining 9% of cows were contaminated. Non-haemolytic staphylococci and α haemolytic streptococci were the pathogens most frequently isolated (Table 2).

**Table 2:** Isolation rates of pathogenic bacteria from positive milk samples^*^

	**Staphylococci**		**Entero-cocci**	**Streptococci**		**Entero-bacteria**		**Bacilli**
**Number of cows**	bH^**^	**NonH**	**αH Esc +** ^**^	**αH Esc-**	**βH**	**Lac +** ^**^	**Lac-**	**βH**
6	X							
28		X						
6			X					
26				X				
1	X	X						
1	X			X				
3		X	X					
5		X		X				
8			X	X				
1			X			X		
1	X	X	X					
3	X		X	X				
4		X	X	X				
1		X				X	X	
1			X	X	X			
1	X	X	X				X	X
**N positive samples**	13	44	28	48	1	2	2	1

^*^see M&M for decisional criteria (presence of strict anaerobes and mycoplasmas was not assessed); ^**^H = haemolytic; Esc = hydrolysis of esculin on Edwards agar; Lac = fermentation of lactose on Gassner agar.

Global fit indices were high for all three SEM, with RMSEA < 0.01, and GFI and NFI > 0.95. More than 95% of the variance in observed CFU, SCC and MILK was explained by the variance in their corresponding latent variable CFU*, SCC* and MILK* (measurement model). Unstandardized estimates of direct effects (elements of **B**) are given in Table 3 for all SEM: direct effects on MILK* were negative for both CFU* and SCC* but significantly (p < 0.05) different from 0 only for SCC*. The indirect effect of CFU* on MILK*, mediated through its effect on SCC*, was −0.50 (SE = 0.37) but was also not significantly different from 0. The total effect was estimated at −3.43 (SE = 2.12).

**Table 3:** Maximum likelihood estimates (and their standard errors) of the direct effects of CFU* and SCC* on MILK* in the three structural equation models (a, b, c)

**Models**	**(a)**		**(b)**		**(c)**	
	unrelated SCC*, CFU*		SCC* influence CFU*		CFU* influence SCC*	
	CFU*	SCC*	CFU*	SCC*	CFU*	SCC*
CFU*	n.a.	n.a.	n.a.	0.12 (0.11)	n.a.	n.a.
SCC*	n.a.	n.a.	n.a.	n.a.	0.05 (0.06)	n.a.
MILK*	−3.08 (1.72)	−9.82^**^ (2.61)	−2.91 (1.74)	−9.78^**^ (2.62)	−2.93 (0.74)	−9.78^**^ (2.62)

n.a. = non applicable; CFU*, SCC* and MILK* = latent variables for log-transformed CFU, log-transformed SCC, and milk yield, respectively; ^**^p < 0.05.

## Discussion

We presented SEM (1) to identify herd characteristics directly related to the risk of infection by mastitis pathogens and (2) to evaluate direct and indirect levels of tolerance of cows from these herds. The SEM approach has several advantages compared to classical methods, such as analysis of variance or regression. Advantages include greater abilities (1) to model complex patterns of relationships or differences between variables, (2) to generate latent variables and reduce measurement error, (3) to provide fit indices for the overall model in addition to individual coefficients, (4) to give a transparent representation of the assumptions built into the model, and (5) to develop mediating variables in addition to variables restricted to an additive model [13]. As such, SEM are useful tools to dissect resistance and tolerance to infectious diseases, and to define accurately the phenotypes corresponding to selection objectives.

In the first part of our study, SEM treated six observed measures of mastitis as imperfect indicators of mastitis status at the herd level (MAM). Therefore, it provided a more accurate assessment of mastitis status than each observed variable taken separately. These six variables were the only ones available in our study but other ones could also be considered, such as the number of cows clinically ill or the herd average California Mastitis Test. Next, we used SEM to evaluate associations between herd characteristics and MAM. We found that nine out of 35 practices were directly and significantly related to MAM, and that four had standardized effects on MAM greater than 0.10. Other management practices had less importance because they either affected indirectly MAM or had direct standardized effects lower than 0.10. Therefore, one may suggest that ‘high-risk’ herds are herds (in our Walloon region) in which cows are pre- but not post-dipped, urea is added in the rations and liners are dirty. For selection purposes, the search for animals that are inherently resistant to infection should be restricted to this type of ‘high-risk’ herds because only in these herds can we be confident that uninfected cows are resistant to direct infection with mastitis pathogens. In other herds, uninfected cows may indeed be resistant but they can also be susceptible and not in contact with mastitis pathogens.

In the second part of the study, we observed a high variability in the number of CFU in infected cows (SE = 98 940 CFU/mL) suggesting that cows may differ in their levels of indirect resistance to mastitis pathogens. We also observed that losses in MILK* through direct and indirect association with CFU* were not significantly different from 0, suggesting that cows were both directly and indirectly tolerant (Table 3). Mastitis could thus spread within the herds because tolerance prolongs the survival of infected hosts, and thus of their pathogens, and this increases the risk of infection for both tolerant and non-tolerant hosts [14]. However, it should be noted that sample size and design of this study were not optimal to detect loss of milk related to presence of bacteria. In theory, bacteria belonging to *Streptococcus* and *Staphylococcus* genera may directly damage tissues by producing several virulence factors and survive within mammary epithelial cells for extended periods of time without losing viability. These bacteria may also cause indirect harm because some of their cell wall-associated and secreted proteins are inflammatory [15].

If infected cows are indeed tolerant and with different levels of indirect resistance, one might wish to include indirect resistance in breeding objectives. This means that one should select cows in which phagocytes are mobilized efficiently from the blood to the udder. Unfortunately, such mobilization (and collateral increase in SCC) was accompanied by a loss estimated at around 9.8 units of MILK* per unit of SCC* (Table 3). Therefore, selection for increased indirect resistance to mastitis (by increasing the number of SCC* per CFU*) would lead to cows that are not tolerant when infected. This suggests that selection should aim at better resistance to infection by mastitis pathogens but further investigations are needed given the limitations of the design of the current study.

Reversely to being advantageous, SEM also have some limits. They are only confirmatory in the sense that theory and design of the study drive the development of the model, as opposed to using data mining to develop a model [16]. Here, designs of both studies were cross-sectional and observational in nature (all variables were measured simultaneously), so no causal inference could be made. Fit indices (RMSEA, GFI, NFI) were similar for the three SEM proposed in the second study and no causal direction between CFU*, SCC* and MILK* could be revealed. Therefore, SEM with other plausible configuration might match the data just as well as the SEM proposed here. Also, sample sizes need to be large enough to provide stable estimates of the parameters: a simplest rule of thumb states that sample sizes of 200 should provide sufficient statistical power [17]. This is lower than the sample size of our study but, given the limitations of its design, further investigations are necessary to validate our results and to better define direct and indirect effects (e.g., non-linear relationships) of tolerance and resistance (e.g., use of a temporal design; correction for moderators; large sample size; use of genetic lines or herds characterized for the risk of infection) to different bacterial species.

## Conclusions

This study described structural equation models with latent variables: (1) to assess the risk for an animal of being exposed to mastitis pathogens and (2) to evaluate direct and indirect levels of tolerance to mastitis. Using this method, we identified nine major risk factors directly associated with an increased risk of mastitis and suggested that cows were tolerant but not resistant to mastitis. The methodology can easily be generalized to other diseases and populations.

## Competing interests

The authors declare that they have no competing interests.

## Authors’ contributions

LT, CH, CB, ER, JM participated in the design and coordination of the study; JND performed the bacteriological analyses; VP selected the farms to be surveyed; CD, MFH performed the survey; JD conceived and coordinated the second study, performed all statistical analyses and drafted the manuscript. All authors have read and approved the final manuscript.
